# Active futures: combating youth sedentary lifestyles in Pakistan through smart use of fragmented time

**DOI:** 10.3389/fspor.2025.1518884

**Published:** 2025-04-08

**Authors:** Sumaira Aslam, Shi Yong Bin

**Affiliations:** School of Physical Education, Henan University Kaifeng, Kaifeng, Henan, China

**Keywords:** fragmented time, sedentary behavior, physical activity, youth, Pakistan, intervention, health promotion

## Abstract

Sedentary behavior among adolescents is a growing concern globally, including in Pakistan, due to its association with negative physical and psychological health outcomes. Fragmented time agility training has arisen as a prospective solution to tackle these challenges. The proposed work evaluates the impact of a structured six-week agility training protocol on sedentary behavior, mood, and stress among adolescents aged 12–18 in Pakistan. A total of 100 participants (50 intervention, 50 control) were recruited from three schools in Islamabad, with equal representation of males and females. Participants were randomly assigned to an intervention group that completed daily 30-min agility training sessions (including high-knee running, lateral shuffles, and cone drills, five days a week) or a control group that maintained their usual routines. Sedentary behavior (sitting and screen time), mood [Profile of Mood States (POMS)], and stress levels [Perceived Stress Scale (PSS)] were assessed at baseline and post-intervention. Differences between groups were analyzed using independent *t*-tests. At post-intervention, significant between-group differences were observed. The intervention group showed reductions in sitting time (6–4 h/day, *p* < 0.001) and screen time (4–2.5 h/day, *p* < 0.001) equated to the control group. Mood improvements were noted with decreased tension and fatigue and increased vigor (*p* < 0.01 for all). Stress levels in the intervention group decreased significantly from 31 to 24 (*p* < 0.001), though no notable alterations were detected in the control group. Concise, systematically organized agility-oriented training significantly diminishes sedentary behavior while concurrently improving psychological well-being in adolescent populations. This adaptable and economically feasible intervention provides critical insights for public health initiatives focused on mitigating sedentary lifestyles and addressing mental health issues among young individuals, especially in contexts with limited resources.

## 1 Introduction

The modern lifestyle has significantly contributed to the prevalence of sedentary behavior, particularly among youth, posing severe health risks. Sedentary behavior is defined as sitting, lying, or reclining with low energy expenditure, such as engaging in screen-based activities like watching television, playing video games, or using a computer. This behavior is distinct from physical inactivity, which refers to not achieving recommended levels of moderate-to-vigorous physical activity (MVPA) (1). Sedentary behavior among youth is a significant public health concern, particularly in developing countries like Pakistan, where lifestyle changes are contributing to rising rates of obesity, cardiovascular diseases, and mental health issues (2). Sedentary behavior, characterized by prolonged sitting, has been linked to adverse cardiovascular and metabolic outcomes, including increased risks for type 2 diabetes, cardiovascular disease, and certain cancers (3).

In Pakistan, a country undergoing rapid urbanization and technological advancement, sedentary lifestyles among young individuals have become alarming. Studies indicate that a substantial portion of Pakistani youth engage in minimal physical activity, with prolonged periods of sitting and screen-based activities. Emerging data suggests that approximately 58.7% of the population in Pakistan leads a sedentary lifestyle, with many adolescents exceeding 4 h of screen time daily for entertainment and educational purposes (4, 5). Sedentary behavior in adolescents is significantly linked to a heightened risk of obesity, cardiovascular illnesses, and adverse mental health outcomes (6). Research indicates that sedentary lifestyles contribute to mental health problems, emphasizing the need for active interventions (7). According to the World Health Organization (WHO), more than 80% of adolescents globally fail to meet the recommended 60 min of MVPA per day (6, 8). According to recent studies, teenagers spend a significant amount of their days doing little physical activity and mostly sedentary tasks like studying and using screens (9, 10). In (11) highlights the beneficial effects of physical exercise on emotional control and mental health in teenagers, consistent with findings from the agility training research. Sedentary behavior, defined by low energy expenditure activities like sitting or reclining, has grown more common owing to technology improvements and lifestyle modifications (9). Investigates the epidemiological aspects of sedentary behavior and its worldwide trends. This relationship is complex and multifaceted, as self-efficacy can influence various psychological and behavioral outcomes (12). The study (13) emphasizes the positive effects of physical activity on physical, mental, and social wellbeing. The research indicates that diverse intervention methods, such as exercise initiatives and behavioral modification strategies, can significantly decrease sedentary behavior in students (14).

Addressing these challenges requires innovative strategies that integrate physical activity into daily life, particularly in resource-constrained environments. While previous research has extensively examined the effects of structured or continuous exercise on sedentary behavior and mental well-being, limited studies have explored the role of short, intermittent activity sessions. This study is unique in its investigation of fragmented agility-based training as a low-cost, scalable intervention specifically designed for adolescents in a resource-constrained setting like Pakistan. Unlike prior research that primarily focuses on traditional physical activity programs, this study evaluates a novel approach that integrates short bursts of high-intensity agility exercises within daily routines. By demonstrating how fragmented agility training can effectively reduce sedentary behavior, improve mood, and lower stress levels, this research provides new insights into time-efficient, feasible solutions for promoting adolescent health. Furthermore, this study contributes to the growing body of knowledge by examining the psychological benefits of agility training, an area that remains underexplored in adolescent populations. Fragmented physical activity, which involves short, structured bouts of movement integrated into daily routines, has emerged as a promising approach to mitigate sedentary behavior (15–17). Unlike generalized examples such as “taking stairs”, fragmented agility training incorporates activities like high-knee running, lateral shuffles, and cone drills, designed to reduce sedentary time while enhancing physical and psychological well-being (15, 16, 18–20). Evidence suggests that even small amounts of MVPA accumulated throughout the day can improve cardiovascular fitness, motor competence, and mental health (21, 22). The psychology of physical activity encompasses a broad range of factors, including determinants, well-being outcomes, and interventions (23). The future of physical activity intervention research is poised to expand significantly by incorporating a focus on sedentary behaviors, leveraging technology, and enhancing dissemination strategies (24).

The study aims to explore how fragmented time interventions can be utilized to reduce sedentary behavior and improve health outcomes among Pakistani adolescents. By focusing on small, manageable activity breaks, this research provides a scalable, low-cost solution to combat sedentary behavior and promote active lifestyles in resource-constrained settings (25). Health-promoting physical exercise and sedentary behavior in children and adolescents are crucial areas of emphasis owing to their substantial influence on physical and mental well-being (26). Regular physical activity (PA) has a multifaceted influence on adolescent mental health, including neurobiological and psychological pathways (27).

This study investigates the effectiveness of a fragmented time agility training protocol in reducing sedentary behavior and improving mood and stress among adolescents in Pakistan. By targeting brief and manageable activity sessions, this intervention provides a scalable and low-cost solution for promoting active lifestyles among youth. The findings aim to inform public health initiatives and educational strategies to combat sedentary behavior and its associated health risks in resource-constrained environments.

In Pakistan, school schedules are typically structured with long periods of seated academic learning and limited opportunities for physical activity. Officially, PE is included in the national curriculum as a compulsory subject for grades 1–12, as outlined in the National Curriculum Framework (2023). However, in practice, PE is often deprioritized in favor of academic subjects, particularly in urban private schools (28, 29). The average school day lasts approximately 6–7 h, during which students remain seated for extended periods, with only one or two short breaks. Physical Education (PE) is often not prioritized in the curriculum, with most schools offering only one to two PE sessions per week, each lasting 30–40 min. However, due to space constraints and academic pressure, these sessions are often irregular or replaced by other subjects (30). Consequently, students in Pakistan spend a significant portion of their school day in sedentary behavior, contributing to declining physical fitness and increased stress levels. Given that school settings play a major role in shaping children's activity levels, this study investigates whether integrating short, structured agility-based exercises into the school environment can provide a feasible and scalable solution to reduce sedentary behavior and improve psychological well-being.

In many schools in Pakistan, break times often contribute to increased screen-based sedentary behavior. Limited access to recreational facilities, combined with academic pressure, leads students to engage in passive activities such as social media browsing, gaming, or watching videos on their mobile devices. The widespread accessibility of mobile phones and a lack of designated activity spaces contribute to students spending a substantial portion of their free time in seated screen-based activities. Addressing this behavioral pattern by promoting agility-based movement during breaks provides a practical approach to reducing overall sedentary time, even if classroom sitting remains unavoidable.

### 1.1 Research gap

Despite the wealth of information regarding sedentary behavior and physical activity, significant gaps remain in the existing research, especially concerning localized studies on Pakistani youth. Although a few studies have highlighted trends in physical activity, comprehensive meta-analyses dedicated specifically to this demographic are lacking (31). Aligning local research on sedentary behavior and physical activity patterns with global findings is essential for fostering effective public health strategies.

While many studies, such as those by Santos-Parker et al. and Luo et al. (32, 33) have highlighted the advantages of physical activity on reasoning function and health, these studies largely focus on continuous or structured exercise rather than fragmented time sessions. Previous research as well proposes that physical activity can improve overall well-being (34–36). However, the current study expands on this by demonstrating how fragmented agility-based training interventions, integrated into daily schedules, can significantly reduce sedentary behavior, perceived stress, and improve mood in adolescents (35–38).

Thus, this study aims to:

1. Assess the impact of fragmented time agility training on sedentary behavior reduction among Pakistani adolescents.
2. Evaluate its effectiveness in improving psychological well-being, including mood and stress levels.
3. Propose a scalable, low-cost intervention that can be integrated into public health strategies for youth in resource-limited settings.

### 1.2 Literature review

Literature on physical activity interventions reveals that numerous research emphasizes the advantages of diverse exercise modes in enhancing both physical and mental health. Santos-Parker et al. investigated the impact of aerobic exercise on vascular and cognitive health, revealing substantial enhancements in both domains (35). Luo et al. similarly found that agility training significantly influences motor competence, reinforcing the notion that physical activity interventions can improve motor skills and coordination (36). Review studies, such as those by Bento et al. and Carson et al., have consolidated overarching trends, demonstrating that metropolitan spatial surroundings and sedentary behavior are associated with substantial health effects, encompassing cardiovascular risk and psychosocial well-being (10, 39). Studies by Russell Jago et al. and Atalla et al. emphasize the significance of physical activity in diminishing sedentary behavior, revealing that educational programs are successful in addressing inactivity, especially among young individuals (37, 38). This study expands on previous findings by investigating the effects of fragmented agility training, which integrates brief intervals of physical activity into daily routines, offering a practical approach to diminish sedentary behavior while enhancing mental and physical health outcomes.

## 2 Methodology

### 2.1 Study design

This research applied a quasi-experimental design to assess impact of utilizing fragmented time for physical activity among Pakistani youth and to measure the effectiveness of agility training intervention to improve sedentary behavior among adolescents aged 12–18. The design compares two groups an intervention group that participates in agility training and a control group that does not receive any intervention. The assessment involves pre-test and post-test measurements to determine the changes in sedentary behavior, physical activity levels, mood, and stress.

### 2.2 Participants and settings

The study involved 100 adolescents aged 12–18 years, recruited from three schools in Islamabad, Pakistan. Schools were chosen based on their willingness to incorporate the intervention during regular school hours. To participate, students needed to meet specific eligibility criteria: they could not have any medical conditions that would restrict physical activity, could not already be part of similar programs, and had to provide parental consent. Participants were randomly divided into two equal groups: 50 in the intervention group and 50 in the control group, with balanced representation of both genders to promote diversity. The intervention was seamlessly integrated into the school schedule, either during physical education classes or designated breaks, ensuring minimal interference with academic activities.

### 2.3 Intervention design

The intervention consisted of 30-min agility training sessions, five days a week for six weeks, structured into a five-minute warm-up, a 20-min agility session, and a five-minute cool-down. The intervention was implemented during PE lessons to ensure structured integration within the school schedule, minimizing disruptions to academic learning. Unlike regular PE classes, which may involve general physical activities, free play, or sports-based games, this agility-based protocol was designed as a structured and time-efficient intervention to specifically target sedentary behavior reduction. The agility training ensured that students consistently engaged in short-duration, high-intensity movements that promote neuromuscular engagement, cardiovascular activation, and cognitive stimulation. Unlike traditional PE classes, which may not consistently emphasize reducing sedentary behavior, this intervention directly counteracted prolonged sitting periods by providing structured agility-based movement. Additionally, the feasibility of integrating this approach into existing PE lessons makes it a scalable and adaptable model for schools, ensuring accessibility without requiring additional school time or resources. Agility exercises included high-knee running, lateral shuffles, cone drills, zigzag runs, and jumping jacks, chosen for their benefits in cardiovascular fitness, coordination, and reaction time. Sessions ended with static stretching and breathing exercises to aid recovery.

Physical activity intensity was measured using the Rate of Perceived Exertion (RPE) scale (6–20 scale), supplemented by heart rate monitoring in a subset of participants (*n* = 20). Activities were classified as moderate-to-vigorous physical activity (MVPA) using established heart rate thresholds (64%–76% HRmax for moderate; >77% HRmax for vigorous intensity). Agility exercises were selected based on evidence supporting their role in improving neuromuscular responses and reducing sedentary behavior (21, 36).

The intervention specifically targeted screen time reduction during school breaks as a strategy to counteract sedentary behavior. Breaks are key periods when students engage in unstructured screen-based activities such as smartphone use, social media browsing, and gaming. By replacing these passive activities with agility-based movement, the intervention aimed to encourage active behavior patterns that could contribute to reducing overall sedentary time throughout the school day. While classroom sitting time remained unavoidable due to academic schedules, promoting movement during breaks provided an opportunity to interrupt prolonged sedentary behavior and reinforce an active lifestyle.

This study introduces a novel approach to reducing sedentary behavior through fragmented agility training, an underexplored method integrating short, high-intensity movement bursts into daily schedules. Unlike conventional structured workouts, this model allows adolescents to accumulate physical activity in a time-efficient manner, making it scalable and adaptable for schools, community settings, and home environments, particularly in resource-limited regions. By evaluating both behavioral (sedentary reduction) and psychological (mood and stress improvement) effects, this study provides new insights into feasible, low-cost strategies for adolescent health promotion.

### 2.4 Intervention implementation

To ensure full engagement in the agility training sessions, mobile phone use was strictly prohibited during all intervention activities. Instructors and PE staff actively monitored the sessions, ensuring that participants remained fully engaged without distractions from screen-based devices. This measure helped maintain the integrity of the intervention and ensured that participants maximized their physical activity levels during the designated training periods.

### 2.5 Behavior change strategies

In order to encourage physical exercise habits, the intervention used behaviour modification strategies. We held weekly 10-min educational seminars to increase awareness of the significance of being active and the health risks linked to extended periods of inactivity. They offered helpful advice on how to properly regulate screen time and incorporate activity into everyday activities. To improve behaviour reinforcement, motivational strategies including goal-setting and self-monitoring (via activity logs) were also used. Additionally, parents in both the intervention and control groups received educational pamphlets emphasizing the importance of reducing screen time and promoting physical activity for their children.

### 2.6 Targeting screen time reduction

By replacing leisure time spent on screens with organized physical activity, the intervention sought to decrease screen time. In order to diminish screen time in the classroom, sessions were scheduled around the times of day when students spend the most time sitting, such as breaks or free periods.

### 2.7 Activity schedule

High-knee jogging in place, lateral shuffles, cone exercises, jumping jacks, and fast foot drills were all part of the agility training. Participants trained for 30 min every day, divided into several sessions. Every session was painstakingly organized, and the table below details the activities and flow.

Table 1 provides a comprehensive breakdown of the activities included in the agility training sessions, detailing the duration and frequency of each exercise to ensure the total session length of 30 min per day.

**Table 1:** Detailed schedule of agility training activities*.*

Activity	Duration per session	Number of sessions per day	Total daily duration (min)
High-knee running in place	1 min	4	4
Lateral shuffles	2 min	4	8
Cone drills	3 min	2	6
Jumping jacks	1–2 min	4	8
Quick feet drills	2 min	2	4

### 2.8 Data collection instruments

Both before (pre-test) and after (post-test) the intervention, data were gathered. demographic survey that asks about history of physical activity, gender, and age. Self-report questionnaires assessing average daily sitting time and screen time (hours per day) were used to measure sedentary behavior. Minutes of exercise per day were used to quantify physical activity. Minutes of physical exercise each day were tracked. Physical activity was tracked using self-reported home-based logs maintained by adolescents and during sports classes held three days a week (50 min per session). The International Physical Activity Questionnaire (IPAQ) was used to assess weekly activity levels, showing an increase from 150 min per week (pre-test) to 170 min per week (post-test), reflecting a 20-min improvement. The standardized Profile of Mood States (POMS) instrument, which monitored changes in tension, weariness, and vigor, was used to measure mood shifts. A comprehensive data collection approach was used to accurately assess the efficacy of the intervention, ensuring reliable measurement of physical activity, sedentary behavior, and psychological outcomes. With Cronbach's alpha values above 0.90 for each of its subscales, the Profile of Mood States (POMS) showed excellent internal consistency, guaranteeing its dependability in identifying mood swings. Cronbach's alpha scores for the Perceived Stress Scale (PSS) range from 0.84 to 0.86, indicating high reliability as well.

### 2.9 Data analysis

The quantitative data from the surveys were analyzed using SPSS (Version 29). Descriptive statistics were used to encapsulate the demographic attributes, physical activity levels, and sedentary behaviors. Paired *t*-tests were used to examine changes in sedentary behavior and physical activity before and after the intervention. Independent *t*-tests were used to assess changes between the intervention and control groups after the intervention.

### 2.10 Ethical considerations

This study was conducted in accordance with the ethical principles of the Declaration of Helsinki and received formal ethical approval from the Bio-Medical Research Ethics Subcommittee of Henan University, China (Approval No. HUSOM2025-465, March 13, 2025). Administrative permission for data collection in Pakistan was granted by Islamabad Career College (Reference No.1109, June 15, 2024). Written informed consent was obtained from participants, with explicit emphasis on voluntary participation, anonymity, and the right to withdraw without consequences. Data confidentiality was rigorously maintained, and protocols minimized risks through supervised age-appropriate agility training.

## 3 Results & findings

### 3.1 Demographic of participants

The study included 100 participants aged 12–18, with an equal gender distribution in both control and intervention groups (Table 2). Participants aged 12–14 were 42% and between 15 and 18 were 58%.

**Table 2:** Demographic of participants (age and gender)*.*

Category	Control group (*n* = 50)	Intervention group (*n* = 50)
Age (12–14)	20	22
Age (15–18)	30	28
Male	25	27
Female	25	23

Analysis of gender differences showed that both boys and girls participants experienced significant reductions in sedentary behavior and improvements in psychological well-being. However, no statistically significant gender-based differences were observed in the intervention outcomes, suggesting that the agility-based training was equally effective across genders.

The physical activities were designed to fit fragmented time sessions of no more than 30 min per day. The exercises, including high-knee running place, lateral shuffles, and cone drills, were divided into multiple sessions, ensuring they could be easily integrated into the daily routines of adolescents (Table 3). This demonstrates the feasibility of the protocol for reducing sedentary behavior within limited constraints.

**Table 3:** Type and duration of the activities*.*

Activity type	Duration per session	Sessions per day	Total duration (mins/day)
High-knee running in place	1 min	4	4 min
Lateral shuffles	2 min	4	8 min
Cone drills	3 min	2	6 min
Jumping jacks	1–2 min	4	8 min
Quick feet drills	2 min	2	4 min
Total			30 min/day

A paired *t*-test was conducted to compare the average sitting time and screen time between the pre-test and post-test scores for both groups. The intervention group showed a significant reduction in sitting time from 6 h to 4 h [*mean difference* = 2.0, *SD* = 0.7, 95% CI: 1.8–2.2, *t* (49) = 4.56, *p* < 0.001], while the control group had no substantial variation [from 6 h to 5.8 h, mean difference = 0.2, SD = 0.6, 95% CI: −0.1–0.5, *t* (49) = 0.75, *p* = 0.46] See (Table 4).

**Table 4:** Pre-test and post-test interventions*.*

Category	Control group (pre-test)	Control group (post-test)	Intervention group (pre-test)	Intervention group (post-test)	*p*-value
Sitting Time (h)	6.0	5.8	6.0	4.0	<0.001
Screen Time (h)	4.0	4.8	4.0	2.5	<0.001

The intervention group showed notable improvements in mood, as measured by the Profile of Mood States (POMS) Tension scores reduced from high to moderate [*t* (49) = 3.12, *p* = 0.003], fatigue also decreased from high to moderate [*t* (49) = 3.67, *p* < 0.001], and vigor levels increased from low to moderate [*t* (49) = 3.45, *p* = 0.001]. In distinction, the control group showed no noteworthy changes in these mood metrics, as shown in Table 5. These findings indicate that short bursts of physical activity positively impact mood regulation, reduce stress, and improve overall mental well-being. ś

**Table 5:** POMS metrics.

Mood metric	Control group (pre-test)	Control group (post-test)	Intervention group (pre-test)	Intervention group (post-test)	*P*-value
Tension	High	High	High	Moderate	0.003
Fatigue	High	High	High	Moderate	<0.001
Vigor	Low	Low	Low	Moderate	0.001

### 3.2 Stress level changes (perceived stress scale)

Stress levels, as assessed by the Perceived Stress Scale (PSS), dropped significantly in the intervention group from a pre-test score of 31 to a post-test score of 24 [mean difference = 7.0, SD = 1.5, 95% CI: 6.5–7.5, *t* (49) = 5.12, *p* < 0.001]. In contrast, the control group showed a minimal change, decreasing from 30 to 29, which was not statistically significant [mean difference = 1.0, SD = 1.2, 95% CI: 0.7–1.3, *t* (49) = 1.23, *p* = 0.22]. These findings highlight the effectiveness of the agility-based intervention in reducing perceived stress compared to usual routines.

**Figure 1:**
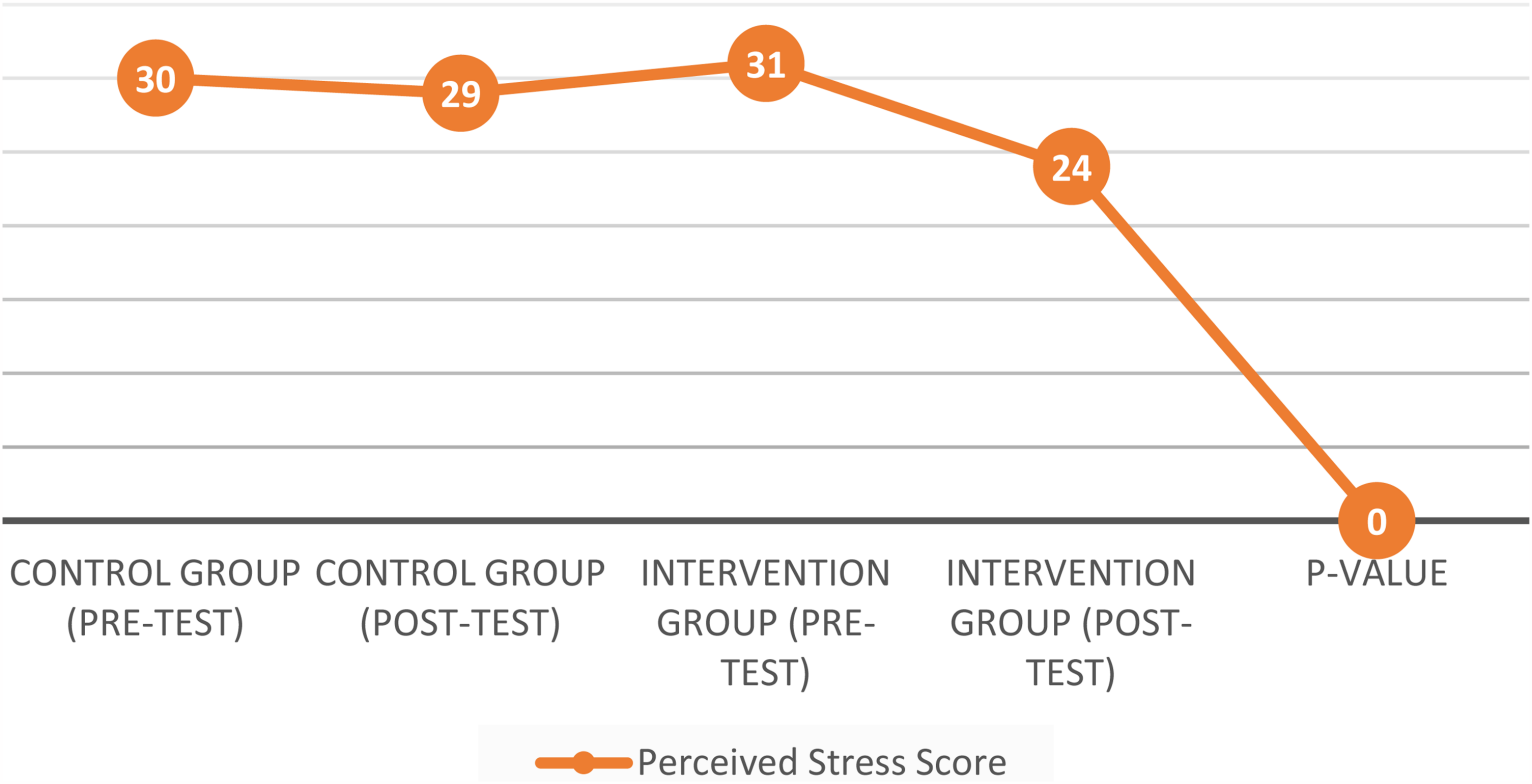
Perceived stress score pre-test & post-test interventions.

Stress levels, as measured by the Perceived Stress Scale (PSS), showed a significant reduction in the intervention group, with scores decreasing from 31 before the intervention to 24 afterward [*t*(49) = 5.12, *p* < 0.001]. In comparison, the control group experienced only a slight drop, from 30 to 29, which was not statistically significant [*t*(49) = 1.23, *p* = 0.22]. The agility-based intervention, tailored to fit fragmented time, proves effective for stress reduction (Figure 1). The *P*-value indicates the statistical significance of the observed reduction in stress levels, reinforcing the effectiveness of the agility-based intervention.

**Figure 2:**
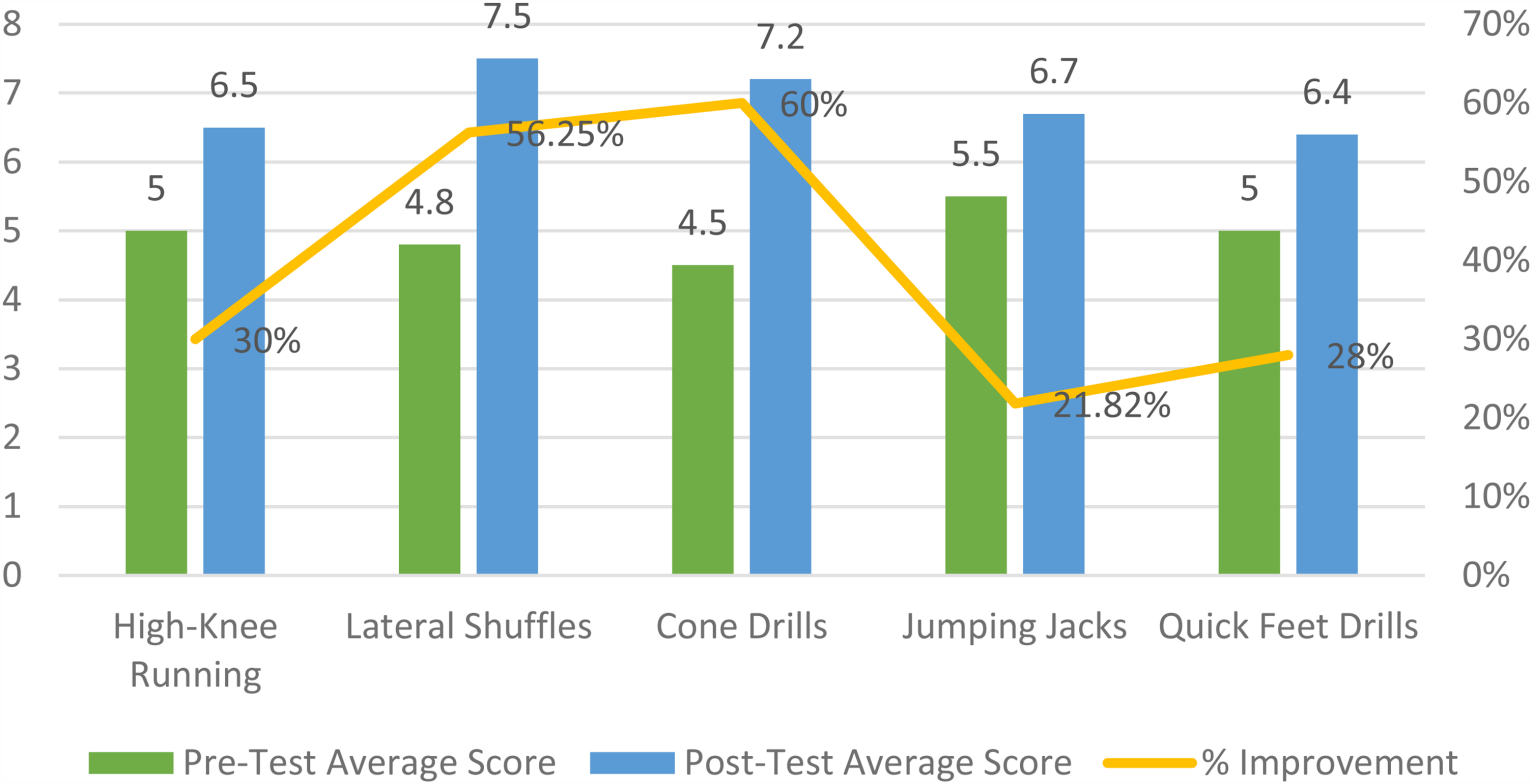
Physical activity improvement pre-test & post-test interventions.

Based on Figure 2. of agility training, lateral shuffles and cone drills (60%) stand out as the most effective exercise (56.25%) for improving overall exercise ability. These activities emphasize agility, coordination, and balance, which are crucial for enhancing physical fitness and motor skills. Lateral movements are essential for agility and dynamic stability, and this exercise improves coordination, quickness, and lower body strength. Cone drills improve footwork, speed, and reflexes, making them ideal for increasing exercise ability in a fragmented training schedule.

**Figure 3:**
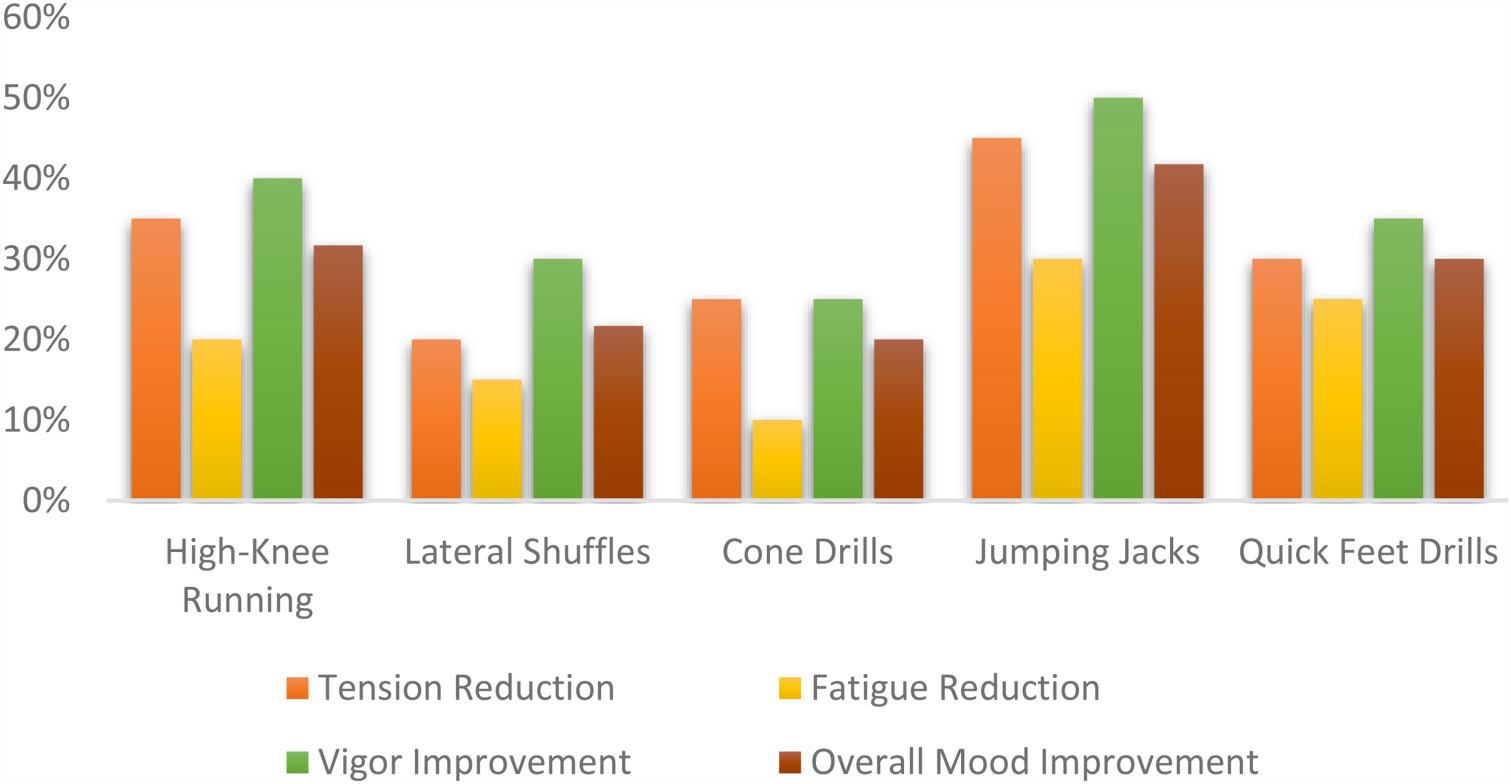
POMS pre-test vs. post-test.

According to the Figure 3. Jumping jacks 41.75% and high-knee running in place 31.67% were most effective in adjusting participants' moods. These exercises involve rhythmic, full-body movements, which stimulate cardiovascular activity and help release endorphins, leading to mood improvement. Jumping jacks are known for releasing endorphins quickly, which helps in reducing stress and improving vigor. High-knee running in Place acts as an aerobic exercise that enhances mood through physical engagement and quick bursts of activity.

**Figure 4:**
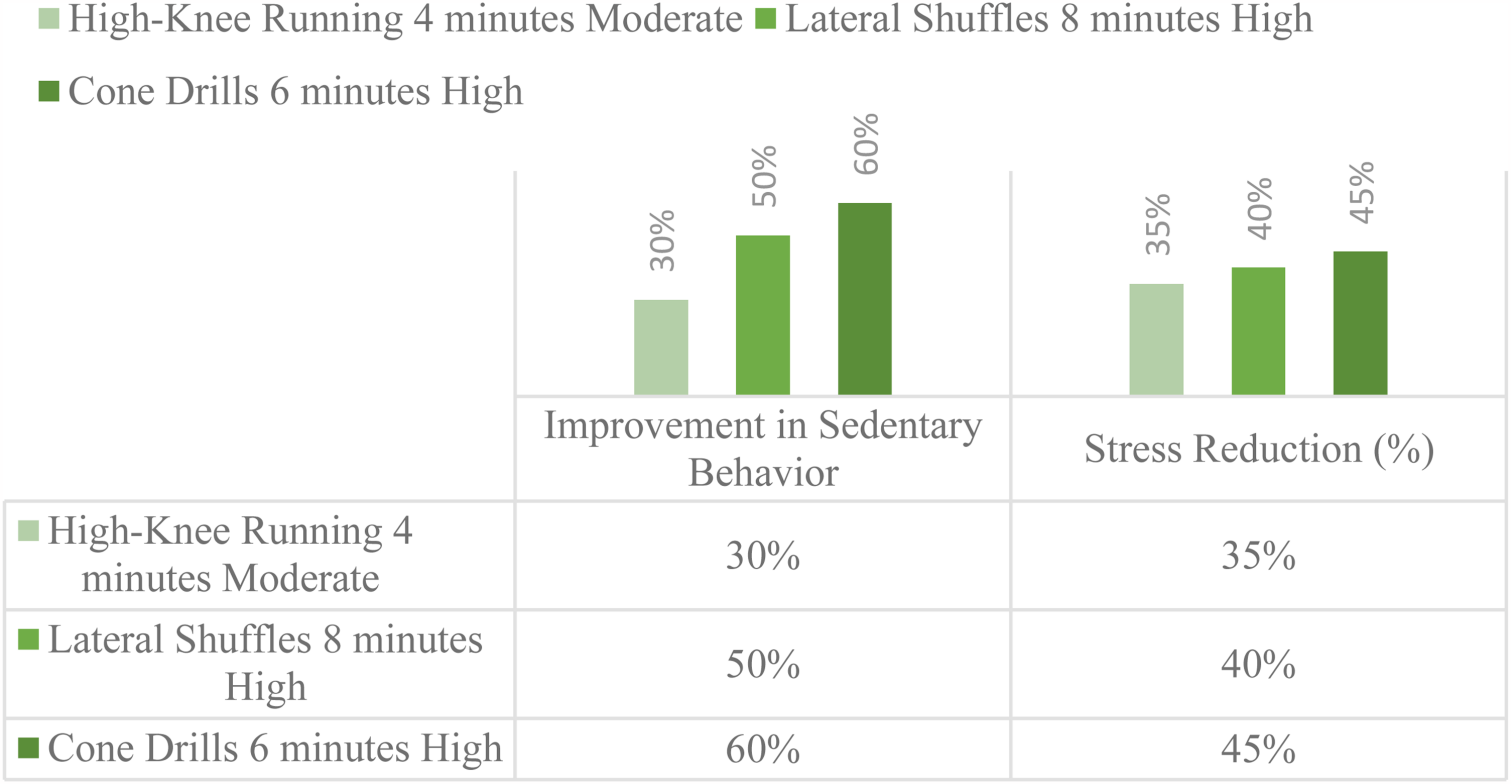
Optimal duration and intensity for fragmented training.

The data suggests that 4–8 min per session is an effective duration for fragmented training (Figure 4). Higher-intensity activities like lateral shuffles and cone drills seem to be more effective in reducing sedentary behavior and stress as compared to moderate-intensity exercises like high-knee running. Overall, this indicates that even short bursts of physical activity can have a positive impact. A combination of moderate and high-intensity exercise might be optimal for achieving the best results.

## 4 Discussion

This study provides novel evidence demonstrating that fragmented agility-based training is an effective, scalable, and time-efficient intervention for reducing sedentary behavior and improving psychological well-being among adolescents. Unlike traditional structured exercise programs, this study is among the first to evaluate short, high-intensity movement bursts as a practical alternative to continuous physical activity, particularly in a resource-limited setting. The findings highlight the effectiveness of integrating agility-based movements into daily routines to combat prolonged sedentary behavior, reduce stress, and enhance mood—offering a feasible and accessible approach for promoting adolescent health.

The results indicate that the intervention led to a statistically significant reduction in sitting time (6–4 h/day, *p* < 0.001) and screen time (4–2.5 h/day, *p* < 0.001). Additionally, the participants in the intervention group exhibited notable mood improvements, including reduced tension and fatigue, alongside increased vigor (*p* < 0.01 for all). Stress levels also decreased significantly, with Perceived Stress Scale (PSS) scores dropping from 31 to 24 (*p* < 0.001). These findings confirm the effectiveness of fragmented agility-based training in enhancing both physical activity levels and psychological well-being, supporting prior research on the role of intermittent movement in mitigating the negative effects of prolonged sitting.

The design of the intervention played a crucial role in achieving these results. The structured short-duration, high-intensity movement bursts ensured that participants could accumulate adequate physical activity without requiring large, uninterrupted time blocks—a key barrier in traditional exercise interventions. The incorporation of agility-based exercises increased neuromuscular engagement, cardiovascular activation, and cognitive stimulation, contributing to the observed improvements in sedentary behavior and psychological health. Furthermore, integrating fragmented training into daily schedules provided a realistic and adaptable solution, reinforcing behavior change without disrupting academic or social routines.

In Pakistan, the proliferation of private schools especially in urban areas has led to a significant disparity in the availability of sports facilities (40). While some elite private schools may have access to well-equipped indoor courts and playgrounds, the reality for the majority of private schools is quite different. Many of these schools, particularly those operating on a commercial model in densely populated cities such as Karachi, Lahore, and Islamabad, lack any form of structured physical education (41). They are often housed in small buildings, with no designated indoor or outdoor spaces for organized sports. This makes it logistically impractical to implement sports such as basketball, futsal, or volleyball, which require dedicated courts and substantial space for safe play.

Additionally, there is a prevailing mindset among many school owners, especially in for-profit private institutions that physical education is not a priority. The academic curriculum is often prioritized to maximize student performance in board examinations, with little to no emphasis on structured physical activity. Even in cases where schools have space, the lack of trained physical education instructors and the reluctance of school administrations to allocate time for sports further limit opportunities for students to engage in physical activities (41).

Given these constraints, agility training presents a uniquely feasible alternative. Unlike traditional sports, agility training does not require large spaces, expensive equipment, or specialized facilities. It can be conducted within classrooms, hallways, or small open areas using minimal resources such as agility ladders, cones, and marker lines. Additionally, agility-based exercises can be seamlessly integrated into physical education lessons without disrupting the school's academic schedule. The structured nature of agility training allows for short, high-intensity bursts of activity, making it practical even in schools where time allocated for physical education is extremely limited.

In addition, agility training offers physiological and cognitive benefits that extend beyond physical fitness. It enhances neuromuscular coordination, reaction time, and proprioception skills which are crucial for overall motor development in children and adolescents (42–44). In a country where childhood obesity and sedentary lifestyles are on the rise due to increased screen time and reduced outdoor play, agility training is an effective intervention to promote physical activity within the existing constraints of private school environments (29).

### 4.1 Practical implications for real-world application

These findings have significant practical implications for schools, community programs, and home-based physical activity strategies. The low-cost, scalable nature of fragmented agility training makes it a viable solution for addressing adolescent sedentary behavior, particularly in resource-limited environments. Schools can incorporate short movement breaks into class schedules, reducing prolonged sitting without compromising instructional time. Community organizations can implement this model in after-school programs or recreational settings to promote engagement in physical activity among youth. Additionally, parents and caregivers can integrate brief agility-based exercises into daily routines at home, reinforcing active lifestyles outside of structured settings.

By demonstrating the effectiveness of short-duration, high-intensity activity bursts, this study provides a practical and accessible intervention model that can be widely adopted in various real-life settings. Future research should explore the long-term sustainability of these benefits, investigate their impact on diverse adolescent populations, and assess potential adaptations for digital or home-based interventions to further enhance accessibility.

The intervention group demonstrated significant reductions in sitting and screen time, showcasing the effectiveness of fragmented agility training in breaking sedentary habits. These findings align with research by Carson et al., which emphasizes the negative effects of prolonged sitting on physical and psychosocial health and highlights the potential of physical activity interventions to mitigate these impacts (10, 35, 45). Physical activity interventions have been increasingly recognized for their potential to improve adolescent mental health (46).

The reduction in sedentary behavior observed in this study is particularly noteworthy given global trends reported via the World Health Organization (WHO), that shows over 80% of adolescents fail to meet recommended physical activity levels (47). By demonstrating a scalable and low-cost intervention that can be seamlessly incorporated into daily routines, this study contributes meaningfully to addressing this global challenge. The structured agility exercises, such as high-knee running, lateral shuffles, and cone drills, were designed to fit within fragmented time, making them feasible for implementation in resource-constrained environments like schools in Pakistan. This aligns with previous research emphasizing that even small increments of physical activity, when accumulated over the day, can significantly improve cardiovascular fitness, motor competence, and mental well-being (21, 47, 48). Physical activity and sedentary behaviors are pivotal in shaping the growth and development of youth, particularly regarding overweight and obesity. Engaging in physical activity is vital for weight management and obesity prevention, whereas sedentary behaviors, including prolonged screen time, lead to weight gain through energy imbalance and poor dietary practices (49, 50). The interplay of physical activity, exercise, and sedentary behavior in college students encompasses various demographic, motivational, and psychological dimensions. A blend of elevated physical activity and sedentary conduct is frequently observed among college students, shaped by factors such as gender, academic standing, and personal motivations (51–55).

Self-reported measures of sedentary behaviour were also cross-validated against trends in the corpus of recent research to increase their consistency and dependability. One of the study's notable findings was the intervention group's participants' emotional states significantly improved. Reduced stress and fatigue as well as increased energy are two benefits of moderate-to-intense physical activity for mental health. These results were supported by the Profile of Mood States (POMS) assessment, a widely used tool for working with adolescent groups. The results align with Santos et al. study, which postulates that short bursts of physical activity may produce endorphins, which lower stress and enhance mental well-being (21, 39). Additionally, the cognitive relief from daily stressors and screen-based activities that these activity intervals offered most likely contributed to mood elevation.

The significant reduction in felt stress levels as measured by the Perceived Stress Scale (PSS) demonstrates the benefits of agility-based training. Being physically and psychologically active during the whole day offers advantages, as seen by the intervention group's average stress level falling by 31–24. It is clear from the observed reduction in stress that other studies have shown that exercise promotes relaxing and reduces cortisol levels (10, 11). Furthermore, the concentration and self-control needed for agility training could have served as a cognitive disruption, helping people to focus on anything other than difficult circumstances. Research done by Saunders et al. This perspective is reinforced by (2021), which emphasizes the importance of initiatives aimed at reducing inactivity and connects sedentary behavior to a higher risk of cardiovascular disease in youth (56).

While the intervention successfully reduced sedentary behavior and improved psychological well-being, it also highlighted the challenges of promoting physical activity in Pakistan. The country's socio-economic and cultural diversity, coupled with rising urbanization and screen-based activities, underscores the need for interventions that are both accessible and culturally adaptable. This study illustrates the feasibility of utilizing fragmented time for physical activity as a practical approach that respects adolescents' time constraints while encouraging active lifestyles. These findings hold particular relevance for low-resource settings, where traditional methods of promoting physical activity may be hindered by financial or infrastructural limitations (47).

This study contributes to the existing research by illustrating the efficacy of fragmented physical activity treatments in diminishing sedentary behavior and improving mental health. The research underscores the advantages of agility-based training, reinforcing the established advantages of moderately to strenuous physical exercise and its significance for various demographics. Future studies should examine the enduring durability of these advantages and assess their relevance across diverse age demographics and cultural settings.

## 5 Strengths, limitations, and future direction

These findings have important implications for schools and public health initiatives. The ease of integrating short, structured agility exercises into daily routines makes this intervention feasible for school settings, helping students reduce sedentary behavior, improve mood, and manage stress. The significant results highlight agility training as a low-cost, scalable intervention for adolescent well-being. Additionally, the flexibility of fragmented time exercises allows implementation in homes, schools, and community centers, supporting its adoption in public health and education policies.

Despite these strengths, some limitations must be acknowledged. The short study duration (6 weeks) limits our ability to assess long-term effects. Additionally, reliance on self-reported data for sedentary behavior and stress may introduce bias. Another limitation is the lack of Public Participatory Involvement (PPI) in intervention design, as participants, educators, and parents were not directly involved. This may have impacted engagement and adherence. Future research should incorporate stakeholder input (via focus groups or pilot studies) to enhance feasibility and participant motivation.

Future studies should also explore the long-term sustainability of agility-based training and its effectiveness in diverse age groups, including younger children and older adults. Additionally, assessing its cognitive and academic benefits could provide broader insights into its role in adolescent development.

## 6 Conclusion

In conclusion, the agility-based fragmented training program significantly reduced sedentary behavior, improved mood, and lowered stress levels in adolescents, as evidenced by the statistically significant. These findings provide strong support for the fragmented time physical activities in promoting the health and well-being of young people. To maximize its impact, this intervention should be systematically integrated into the national Physical Education (PE) curriculum, which currently mandates PE as a compulsory subject but faces systematic implementation challenges. The agility program aligns with the National Education Policy's (2020–23) emphasis on holistic development. To operationalize this, the Ministry of Federal Education and Professional Training could revise curriculum guidelines to designate daily 15–20-min agility sessions as a core component of PE. These sessions comprising high-knee runs, lateral shuffles, and cone drills can be embedded within existing PE classes or breaks, requiring minimal space and equipment. Pilot initiatives in Punjab's secondary schools where PE neglect is pronounced due to exam-centric cultures could serve as a model. Successful pilots may include agility breaks between classes to disrupt prolonged sitting. student-led peer groups to foster accountability and engagement. Provincial education departments should mandate minimum daily physical activity standards (30 min of MVPA), and integrate agility metrics into school performance evaluations. Incorporating standardized fitness testing into school curricula can provide objective metrics to monitor physical activity interventions and tailor programs to student needs, thereby enhancing accountability and long-term health outcomes. Integrating these exercises into daily routines, schools, and communities can help adolescents lead more active and fulfilling lives.

## Data Availability

The original contributions presented in the study are included in the article/Supplementary Material, further inquiries can be directed to the corresponding author.

## References

[1] Tremblay MS LeBlanc AG Kho ME Saunders TJ Larouche R Colley RC Canadian 24-hour movement guidelines for children and youth: an integration of physical activity, sedentary behaviour, and sleep. Appl Physiol Nutr Metab. (2016) 41(6):S311–27. 10.1139/apnm-2016-0151 27306437

[2] Ramírez Varela A Hallal PC Mejía Grueso J Pedišic Ž Salvo D Nguyen A Status and trends of physical activity surveillance, policy, and research in 164 countries: findings from the global observatory for physical activity—GoPA! 2015 and 2020 surveys. J Phys Act Health. (2023) 20(2):112–28. 10.1123/jpah.2022-0464 36535269 PMC10115485

[3] Hamilton MT Healy GN Dunstan DW Zderic TW Owen N. Too little exercise and too much sitting: inactivity physiology and the need for new recommendations on sedentary behavior. Curr Cardiovasc Risk Rep. (2008) 2(4):292–8. 10.1007/s12170-008-0054-8 22905272 PMC3419586

[4] Chaput JP Willumsen J Bull F Chou R Ekelund U Firth J 2020 WHO guidelines on physical activity and sedentary behaviour for children and adolescents aged 5–17 years: summary of the evidence. Int J Behav Nutr Phys Act. (2020) 17(1):141. 10.1186/s12966-020-01037-z 33239009 PMC7691077

[5] Naseer R. Physical inactivity and health. Pak BioMed J. (2022) 5(10):02–02. 10.54393/pbmj.v5i10.816

[6] Bann D Scholes S Fluharty M Shure N. Adolescents’ physical activity: cross-national comparisons of levels, distributions and disparities across 52 countries. Int J Behav Nutr Phys Act. (2019) 16(1):141. 10.1186/s12966-019-0897-z 31888652 PMC6937925

[7] Rahim F Farooq A Irfan MB Ullah R Jamil T Rehman AU From screen to depression: an integrative review link between sedentary lifestyle and mental health issues: sedentary lifestyle and mental health. J Rehabil Res Dev. (2024) 4(3):1–6.

[8] Janssen I LeBlanc AG. Systematic review of the health benefits of physical activity and fitness in school-aged children and youth. Int J Behav Nutr Phys Act. (2010) 7:1–16. 10.1186/1479-5868-7-40 20459784 PMC2885312

[9] Bauman AE Petersen CB Blond K Rangul V Hardy LL. The descriptive epidemiology of sedentary behaviour. In: Leitzmann MF, editor. Sedentary Behaviour Epidemiology. Regensburg: Epidemiology and Preventive Medicine, Universität Regensburg (2018). p. 73–106. 10.1007/978-3-319-61552-3_4

[10] Carson V Hunter S Kuzik N Gray CE Poitras VJ Chaput J-P Systematic review of sedentary behaviour and health indicators in school-aged children and youth: an update. Appl Physiol Nutr Metab. (2016) 41(6):S240–65. 10.1139/apnm-2015-0630 27306432

[11] Martinez AG Gietzen L McDaniel VF. Exploring the role of physical activity influencing emotional regulation and mental health in adolescents. Pac J Health. (2024) 7(1):1.

[12] Cheron G Forte P Forte P Teixeira J Portella D Portella DL Open Access Edited And Reviewed By. (2024). p. 4.

[13] Kemel PN Porter JE Coombs N. Improving youth physical, mental and social health through physical activity: a systematic literature review. Health Promot J Austr. (2022) 33(3):590–601. 10.1002/hpja.553 34735738

[14] Epstein LH Roemmich JN. Reducing sedentary behavior: role in modifying physical activity. Exerc Sport Sci Rev. (2001) 29(3):103–8. 10.1097/00003677-200107000-00003 11474956

[15] Guthold R Stevens GA Riley LM Bull FC. Global trends in insufficient physical activity among adolescents: a pooled analysis of 298 population-based surveys with 1.6 million participants. Lancet Child Adolesc Health. (2020) 4(1):23–35. 10.1016/S2352-4642(19)30323-2 31761562 PMC6919336

[16] Stalsberg R Pedersen AV. Effects of socioeconomic status on the physical activity in adolescents: a systematic review of the evidence. Scand J Med Sci Sports. (2010) 20(3):368–83. 10.1111/j.1600-0838.2009.01047.x 20136763

[17] Mittal VA Firth J Kimhy D. Combating the dangers of sedentary activity on child and adolescent mental health during the time of COVID-19. J Am Acad Child Adolesc Psychiatry. (2020) 59(11):1197. 10.1016/j.jaac.2020.08.003 32860908 PMC7448948

[18] Joensuu L. Longitudinal study on physical fitness characteristics in adolescents with special reference to the determinants of change and associations with perceived health. [JYU dissertations]. (2021).

[19] Contardo Ayala AM Parker K Mazzoli E Lander N Ridgers ND Timperio A Effectiveness of intervention strategies to increase adolescents’ physical activity and reduce sedentary time in secondary school settings, including factors related to implementation: a systematic review and meta-analysis. Sports Med Open. (2024) 10(1):25. 10.1186/s40798-024-00688-7 38472550 PMC10933250

[20] Lüddeckens D Hetmanczyk P Klassen PE Stein JB. The Routledge Handbook of Religion, Medicine and Health. London: Routledge (2022).

[21] Santos B Monteiro D Silva FM Flores G Bento T Duarte-Mendes P. Objectively measured physical activity and sedentary behaviour on cardiovascular risk and health-related quality of life in adults: a systematic review. Healthcare. (2024) 12(18):1866. 10.3390/healthcare12181866 39337207 PMC11431446

[22] Tremblay MS Systematic review of sedentary behaviour and health indicators in school-aged children and youth. Int J Behav Nutr Phys Act. (2011) 8(1):98. 10.1186/1479-5868-8-98 21936895 PMC3186735

[23] Biddle S Mutrie N. Psychology of Physical Activity: Determinants, Well-being and Interventions. London: Routledge (2007).

[24] Lewis BA Napolitano MA Buman MP Williams DM Nigg CR. Future directions in physical activity intervention research: expanding our focus to sedentary behaviors, technology, and dissemination. J Behav Med. (2017) 40:112–26. 10.1007/s10865-016-9797-8 27722907 PMC5296224

[25] Karim Y Manzoor W Zainab SS ur Rahman A Yousaf D. Consequences of screen-based lifestyle among university students: an empirical study based on moderated mediation model in Pakistan. Int J Soc Sci Arch (IJSSA). (2023) 6(3).

[26] Biddle SJ Gorely T Stensel DJ. Health-enhancing physical activity and sedentary behaviour in children and adolescents. J Sports Sci. (2004) 22(8):679–701. 10.1080/02640410410001712412 15370482

[27] Bajwa HA Iqbal MU Ali MS Abbas MA Gul A Ghani M Multidimensional impact of regular physical activity on adolescent mental health, integrating neurobiological and psychosocial mechanisms: exercise reduces depression and anxiety in youth. Dev Med Life Sci. (2024) 1(7):20–8. 10.69750/dmls.01.07.060

[28] Ali K Sadaf A Kousar S Habib Z. Standard of physical education in Pakistan as compare to U.S.A. global journal of human-social science research. Glob J Hum Soc Sci. (2014) 14.

[29] Tanveer M Asghar E Badicu G Tanveer U Roy N Zeba A Associations of school-level factors and school sport facility parameters with overweight and obesity among children and adolescents in Pakistan: an empirical cross-sectional study. Sports (Basel). (2024) 12(9). 39330712 10.3390/sports12090235 PMC11435805

[30] Raza FA. A brief comment on the condition of physical education in schools in Pakistan. Int J Med Sci Health Res. (2021) 12(9).

[31] Imtiaz A ulHaq Z Afaq S Khan MN Gillani B. Prevalence and patterns of physical activity among school aged adolescents in Pakistan: a systematic review and meta-analysis. Int J Adolesc Youth. (2020) 25(1):1036–57. 10.1080/02673843.2020.1831559

[32] Chaudhary A Mahmood S Jamil M Khan A Butt MZI. Physical activity as an element of health life style among high school children’s: an analytical approach: physical activity among high school children’s. Pak J Health Sci. (2022) 3(7):190–4. 10.54393/pjhs.v3i07.372

[33] Di J Leroux A Urbanek J Varadhan R Spira AP Schrack J Patterns of sedentary and active time accumulation are associated with mortality in US adults: The NHANES study. BioRxiv. (2017): p. 182337.

[34] del Pozo-Cruz J del Pozo Cruz B Perez-Sousa MÁ Alfonso-Rosa RM. High fragmented physical activity as an early risk indicator of frailty and mortality in adults aged 50 years and over. Gerontology. (2023) 69(3):370–8. 10.1159/000525910 36481521

[35] Santos-Parker JR LaRocca TJ Seals DR. Aerobic exercise and other healthy lifestyle factors that influence vascular aging. Adv Physiol Educ. (2014) 38(4):296–307. 10.1152/advan.00088.2014 25434012 PMC4315444

[36] Luo X Herold F Ludyga S Gerber M Kamijo K Pontifex MB Association of physical activity and fitness with executive function among preschoolers. Int J Clin Health Psychol. (2023) 23(4). 10.1016/j.ijchp.2023.100400 PMC10469079 37663042

[37] Atalla M Pinto AJ Mielke GI Baciuk EP Benatti FB Gualano B. Tackling youth inactivity and sedentary behavior in an entire Latin America city. Front Pediatr. (2018) 6:298. 10.3389/fped.2018.00298 30370264 PMC6194316

[38] Jago R Fox KR Page AS Brockman R Thompson JL. Physical activity and sedentary behaviour typologies of 10–11 year olds. Int J Behav Nutr Phys Act. (2010) 7(1):59. 10.1186/1479-5868-7-59 20663226 PMC2918527

[39] Bento G Dias GJ. The importance of outdoor play for young children’s healthy development. Porto Biomed J. (2017) 2(5):157–60. 10.1016/j.pbj.2017.03.003 32258612 PMC6806863

[40] Bento G. Plight of physical education in secondary schools of Punjab; Pakistan. Int J Educ Res. (2019) 10(3):11–25.

[41] Saeed N Shah DR Khan DI. A qualitative inquiry into the role and issues of physical education in primary schools: perspectives of primary school teachers. SJESR. (2023) 10(3):11–25.

[42] Morat M Morat T Zijlstra W Donath L. Effects of multimodal agility-like exercise training compared to inactive controls and alternative training on physical performance in older adults: a systematic review and meta-analysis. Eur Rev Aging Phys Act. (2021) 18. 10.1186/s11556-021-00256-y 33632117 PMC7908670

[43] Zech A Hübscher M Vogt L Banzer W Hänsel F Pfeifer K. Balance training for neuromuscular control and performance enhancement: a systematic review. J Athl Train. (2010) 45(4):392–403. 10.4085/1062-6050-45.4.392 20617915 PMC2902034

[44] Francis A Mathew L. Effectiveness of brisk walking on VO2 max, agility and flexibility in physically inactive children. Int J Sci Healthc Res. (2023) 8(2):141–56. 10.52403/ijshr.20230218

[45] van Sluijs EM Ekelund U Crochemore-Silva I Guthold R Ha A Lubans D Physical activity behaviours in adolescence: current evidence and opportunities for intervention. Lancet. (2021) 398(10298):429–42. 10.1016/S0140-6736(21)01259-9 34302767 PMC7612669

[46] Li Z Li J Kong J Li Z Wang R Jiang F. Adolescent mental health interventions: a narrative review of the positive effects of physical activity and implementation strategies. Front Psychol. (2024) 15:1433698. 10.3389/fpsyg.2024.1433698 38993342 PMC11236730

[47] Rizwan M Alam MM Akhtar MW Inam F Yaseen F Alam S Association between sedentary lifestyle and quality of life among young adults. J Health Rehabil Res. (2024) 4(1):774–9.

[48] Caspersen CJ Powell KE Christenson GM. Physical activity, exercise, and physical fitness: definitions and distinctions for health-related research. Public Health Rep. (1985) 100(2):126. 3920711 PMC1424733

[49] Hills AP King NA Armstrong TP. The contribution of physical activity and sedentary behaviours to the growth and development of children and adolescents: implications for overweight and obesity. Sports Med. (2007) 37:533–45. 10.2165/00007256-200737060-00006 17503878

[50] Ortega FB Ruiz JR Castillo MJ Sjöström M. Physical fitness in childhood and adolescence: a powerful marker of health. Int J Obes (Lond). (2008) 32(1):1–11. 10.1038/sj.ijo.0803774 18043605

[51] Buckworth J Nigg CJ. Physical activity, exercise, and sedentary behavior in college students. J Am Coll Health. (2004) 53(1):28–34. 10.3200/JACH.53.1.28-34 15266727

[52] Lee E Kim Y. Effect of university students’ sedentary behavior on stress, anxiety, and depression. Perspect Psychiatr Care. (2018) 55(2):164. 10.1111/ppc.12296 29797324 PMC7818186

[53] Herbert C. Enhancing mental health, well-being and active lifestyles of university students by means of physical activity and exercise research programs. Front Public Health. (2022) 10:849093. 10.3389/fpubh.2022.849093 35548074 PMC9082407

[54] Deliens T Deforche B De Bourdeaudhuij I Clarys P. Determinants of physical activity and sedentary behaviour in university students: a qualitative study using focus group discussions. BMC Public Health. (2015) 15:1–9. 10.1186/s12889-015-1553-4 25881120 PMC4349731

[55] Lavie CJ Ozemek C Carbone S Katzmarzyk PT Blair SN. Sedentary behavior, exercise, and cardiovascular health. Circ Res. (2019) 124(5):799–815. 10.1161/CIRCRESAHA.118.312669 30817262

[56] Prince SA Dempsey PC Reed JL Rubin L Saunders TJ Ta J The effect of sedentary behaviour on cardiorespiratory fitness: a systematic review and meta-analysis. Sports Med. (2024) 54(4):997–1013. 10.1007/s40279-023-01986-y 38225444 PMC11052788

